# Effects of startle on cognitive performance and physiological activity revealed by fNIRS and thermal imaging

**DOI:** 10.1038/s41598-025-90540-z

**Published:** 2025-02-26

**Authors:** Flora Schwartz, Jonathan Deniel, Mickaël Causse

**Affiliations:** 1 Institut Supérieur de l’Aéronautique et de l’Espace – Supaero, Université de Toulouse, Toulouse, France; 2 Institut National Universitaire Champollion, Albi, France

**Keywords:** Auditory startle, Mental workload, fNIRS, fITI, Executive control network, Facial temperature, Cognitive neuroscience, Stress and resilience, Human behaviour

## Abstract

Sudden and threatening stimuli can trigger a startle reflex, a stereotyped physiological response that may lead to a brief cognitive incapacitation. Better understanding this reaction would be beneficial to safety-critical occupational domains. We characterized some physiological correlates of the startle response while participants completed a difficult task (Toulouse N-back task) tapping executive functions. During the task, loud and threatening sounds were presented unpredictably to trigger a startle reflex. Brain activity and facial skin temperature were measured in 34 participants using functional near-infrared spectroscopy (fNIRS) and functional infrared thermal imaging (fITI), respectively. In the high difficulty condition, participants were generally less efficient, but their performance improved slightly following startle in the high difficulty condition. Brain activity in the right prefrontal cortex was also higher following startle, potentially reflecting a compensatory overactivation to sustain performance. Interestingly, higher trait-anxiety was associated with lower task performance, still following startle in the high difficulty condition. Finally, we found a decrease in temperature of the right eye and right cheek as well as an increase in the nose temperature following startle. These results underscore the complexity of startle-induced cognitive and physiological dynamics, which may have implications for occupational settings where managing sudden stressors is crucial.

**Supplementary Information:**

The online version contains supplementary material available at 10.1038/s41598-025-90540-z.

## Introduction

### Human performance in complex environments

Understanding how humans maintain high cognitive performance is critical to accompany individuals in the completion of cognitively demanding tasks. Such endeavor also has implications for designing tools that can monitor and potentially regulate cognitive performance in complex environments. Among the environmental stressors that may impact on cognitive performance, sudden stimuli may induce a startle reflex^1–3^, a physiological reaction characterized by eye blinks, muscle tension in the face and neck and freezing. Depending on context, the startle reflex may involve a startle response that may be augmented by fear or stress^4–6^ and disrupt actions or cognitive operations^7–11^, and even lead to temporary cognitive incapacitation under mental workload and stress^12^. As an example, individuals may show longer response time immediately following auditory startle^11^, or make more erroneous response both during a simple motor inhibition task^7^ or a highly complex task involving a human-machine interface^8,12^. Although the decline in cognitive performance following startle is brief^11,13^, the startle response may thus have dramatic consequences in the transportation and safety domain, such as aircraft piloting^14,15^ with critical decisions to be made sometimes within a few seconds. Hence the growing interest in Neuroergonomics to identify objective markers that can predict the state of cognitive deterioration following startle. Importantly, the literature has documented individual differences in the startle response^16^ and has more specifically associated dispositions toward anxiety with increased behavioral^17–19^ and neural^18,20^ response to startling stimuli. This evidence highlights the need to determine how markers of the startle response are sensitive to individual differences between operators such as susceptibility to stress and personality traits.

The goal of the present study was to investigate the physiological correlates of the startle response during a cognitively demanding task using multi-modal and non-invasive recordings, namely functional near-infrared spectroscopy (fNRIS) and thermal imaging. We had participants perform an N-back task combined with mental arithmetic and determined how brain activity and facial skin temperature changed following an auditory startle, and how such changes were related to task difficulty and performance.

### Brain activity changes following startle

The startle reflex may interfere with ongoing cognitive tasks by impacting brain functioning. Cognitively demanding tasks typically involve the brain’s Executive control network (ECN), which includes the dorsomedial prefrontal cortex (dmPFC), dorsolateral prefrontal cortex (DLPFC), and posterior parietal cortex (PPC). The ECN has been identified as supporting executive functions, such as managing cognitive load, supporting goal-driven attention, and manipulating items in working memory^16,17^. A typical task tapping the ECN is the N-back procedure^18^, requiring individuals to track the identity or position of items and responding whether a given item matches the item presented n trials before.

Activity within the ECN increases with task demand^19–21^, but may also increase under stress for the same cognitive load^22,23^, thus reflecting what are thought of as compensatory mechanisms that help maintain performance. Therefore, tracking ECN activity may indicate not only whether cognitive load is high for individuals, but also how effortful it is to perform the task in different contexts. It may for instance help assess whether operators maintain performance following startle. This information would be valuable for the design of brain-computer interface tools that monitor the cognitive state of operators.

From this perspective, functional near-infrared spectroscopy (fNIRS) is a convenient tool^24–26^ to track changes in mental workload. It uses near-infrared light emitted by optodes on the surface of the skull that penetrates a few centimeters into the brain before being received by the nearest detectors placed on the surface of the skull. Some of the light is absorbed by metabolites (oxy- and deoxy-hemoglobin) whose concentration vary depending on brain functioning. This allows to indirectly track changes in activity in brain regions such as the ECN which are not too deep under the skull. Because fNIRS is non-invasive, easy to use and relatively robust to head motion, this technique can be deployed in more ecological settings in comparison with other brain imaging tools. Many studies have used fNIRS to track ECN activity associated with cognitive demand, for instance during N-back tasks^27–30^. Some fNIRS studies have documented how ECN activity changes under stress during cognitively demanding tasks^23,31^, overall pointing to compensatory overactivation to maintain task performance under stress. How is ECN activity modulated following startle specifically?

To our knowledge, only one fNIRS study on auditory startle has been published. This study used a motor reaching task and measured brain activity in the frontal pole and right DLPFC following a startling sound or a control sound. The authors found reduced HbO-signal in the right DLPFC (as well as motor regions) during hand reaching following an auditory startle^32^. Previous neuroscience studies of startle using EEG or fMRI have mostly manipulated the affective context of the startle stimulus, for instance by relying on a fear conditioning paradigm or by presenting pleasant and unpleasant stimuli. These studies have identified brain networks involved in both the motor response and the sensory response to the conditioned stimulus (see^33^, for a review) and have pointed to greater amplitude of the early negativity and posterior positivity in response to threatening stimuli^34–36^. However, how brain activity changes following startle while individuals are engaged in a cognitively demanding task remains largely unexplored.

### Facial temperature changes following startle

Functional infrared thermal imaging (fITI) enables contact-free measures of skin temperature, which provides information about the functioning of the autonomic nervous system at a given time, thus indirectly assessing changes in affective states^37^. Previous research has linked different emotions, such as fear or guilt, to different patterns of temperature changes in different regions of the face, as reviewed by Ioannou and colleagues. As regards temperature changes following startle specifically, a few studies point to temperature changes in the cheeks, maxillary, periorbital areas, though the direction of the temperature change and exact set of regions somewhat depends on the study^38–42^. For example, auditory startle has been associated with temperature increase in the periorbital areas^40,42^, but not always^43^. Other studies found a decrease in the temperature of the cheeks^40,44^ and nose^44^, or increase in temperature of the maxillary^42^ and forehead^45^. In the present study, we targeted the face regions already identified in this limited set of studies and determined the temperature changes associated with auditory startle.

### The present study

In the present study, we had participants perform a cognitively demanding task which included sound stimuli expected to trigger a startle response (“startle sounds”) as well as control stimuli (“control sounds”). The task featured a low-cognitive load condition (“0-back”) which required participants to respond whether the result of the calculation was true or false, and a high-load condition (“2-back”) which required to respond whether the result matched that of the calculation presented two trials earlier. We expected a temporary performance decline following startle stimuli specifically. Our goal was to investigate the physiological correlates of the startle response using a combination of thermal imaging and fNIRS.

First, we aimed at characterizing activity changes in regions indexing the ECN functioning following startle for different levels of cognitive load using the Toulouse N-back task (TNT), a task combining mental arithmetic with a traditional N-back task (see Methods). We expected activity in the ECN to be higher in the startle condition, especially under high cognitive load.

Second, we applied a facial recognition algorithm to our thermal imaging data and measured temperature changes following startle in a set of face regions identified in the literature. We expected higher temperature variation in the startle condition relative to the control condition.

## Results

### Behavioral results

#### Perceived task demand

**Fig. 1:**
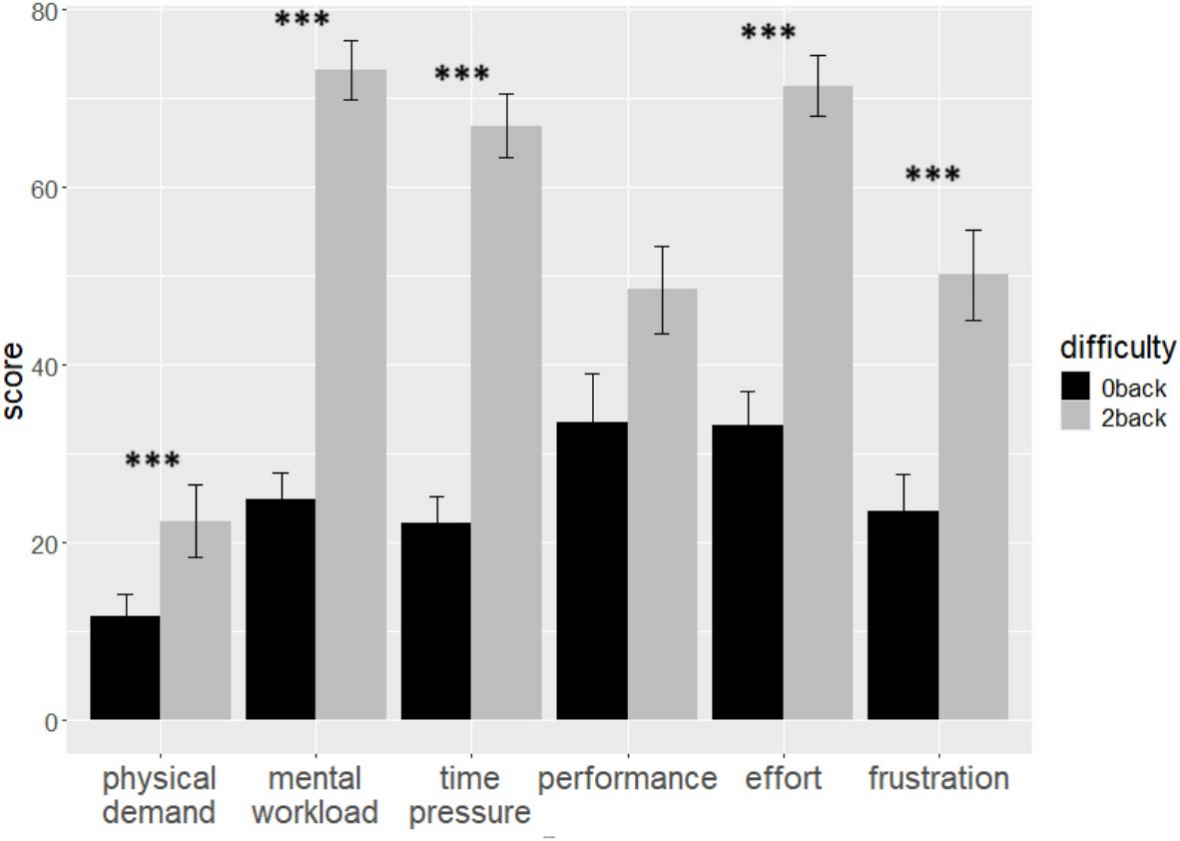
Score at the NASA task load index questionnaire administered after the TNT for each difficulty condition. Error bars represent the standard error of the mean. *** *p* < .001.

We first present participants’ perceived task demand for the two difficulty conditions (Fig. 1) as assessed by the NASA Task load index (see Method). Participants felt overall higher mental workload (*V* = 435, *p* < .001, *Z* = 0.79), time pressure (*V* = 462, *p* < .001, *Z* = 0.76), and frustration (*V* = 412, *p* < .001, *Z* = 0.46) in the 2-back condition relative to the 0-back condition, and additionally reported more effort (*V* = 401.5, *p* < .001, *Z* = 0.69) and physical demand (*V* = 186, *p* < .001, *Z* = 0.21). Self-rated performance failure tended to be higher in the 2-back condition relative to the 0-back condition (*V* = 268, *p* = .058, *Z* = 0.26). The sensitivity power analysis revealed an achieved power superior to 95% for the effect of difficulty on mental workload, time pressure and effort, and an achieved power of 68%, 20% and 27% for the effect of difficulty on frustration, performance and physical demand, respectively.

#### Behavioral observation of startle

To check whether the manipulation induced startle, we relied on a combination of both live observation while participants completed the TNT and aposteriori visualization of the thermal videos. Facial expressions associated with startle were analyzed by observing eye closure, horizontal lip stretch, and head and trunk movements^46,47^. Facial expressions related to surprise were analyzed by observing raised eyebrows, widened eyes, and jaw drop or mouth opening^47,48^. Reaction of startle or surprise was noted as positive if at least one of these observable markers was present. It was found that 65% of the participants (22 out of 34 participants who completed the study, and 18 out of the 27 participants included in the brain activity analysis) showed clear signs of startle immediately following at least one of the sounds.

#### Performance at the TNT

**Fig. 2:**
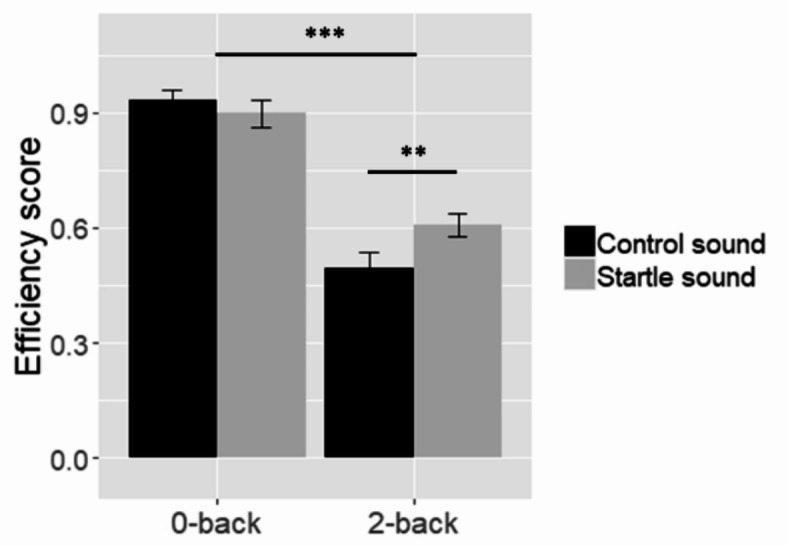
Performance at the TNT. Error bars represent the standard error of the mean. ** *p* < .01; *** *p* < .001.

We computed efficiency based on the mean accuracy and response times at the first three calculations following each sound onset (see method). As expected, difficulty had a significant effect on efficiency at the TNT (*F*(1,33) = 123, *p* < .001, *η*^2^ = 0.46). Participants were more efficient at the 0-back condition (*M* = 0.91, *SD* = 0.18) relative to the 2-back condition (*M* = 0.55, *SD* = 0.22). Efficiency was not significantly different between the startle sound and the control sound (*F*(1,33) = 2.14, *p* = .15, *η*^2^ = 0.01) (startle sound: *M* = 0.75, *SD* = 0.24; control sound: *M* = 0.71, *SD* = 0.30) but the interaction between sound type and difficulty reached significance (*F*(1,33) = 9.8, *p* = .004, *η*^2^ = 0.034). In the 2-back condition, participants were more efficient following startle sounds than control sounds (*t*(33) = -2.96, *p* = .006, *d* = 0.54) while this was not the case in the 0-back condition (*t*(33) = 1.02, *p* = .32, *d* = -0.18). See Fig. 2 below. The sensitivity power analysis revealed an achieved power superior to 95% for the main effect of difficulty on efficiency and of 94% for the interaction between difficulty and sound type. See supplementary results S1 for the analysis of accuracy and response times, separately.

#### Effect of trait-anxiety

**Fig. 3:**
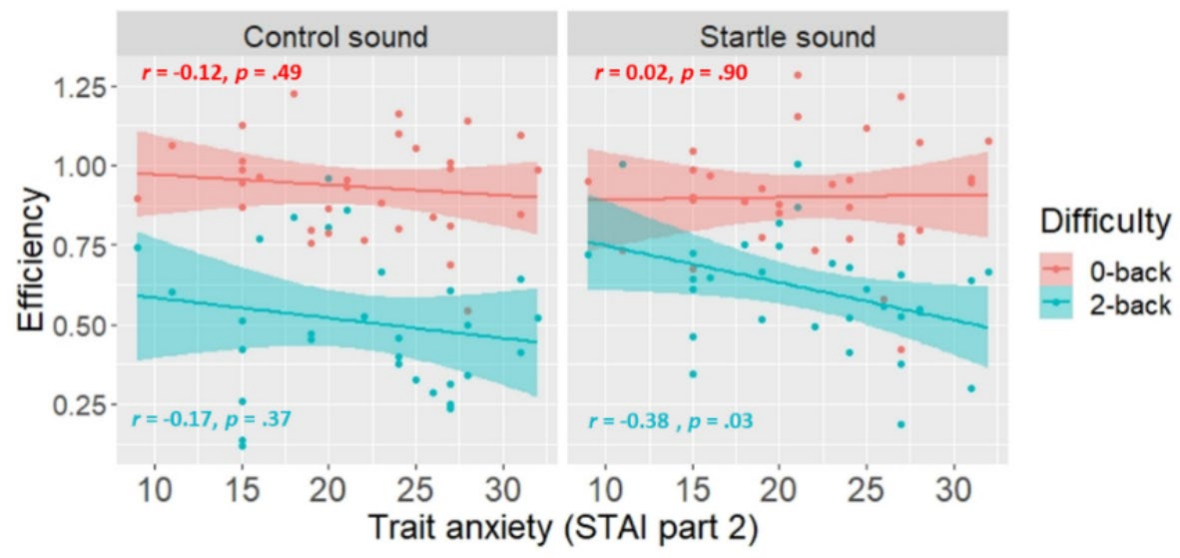
Association between trait anxiety and performance at the TNT. The smooth line represents the 95% confidence interval of the regression line.

In exploratory analyses, we investigated the association between personality variables and performance at the TNT. More specifically, we focused on state- and trait-anxiety (measured with the STAI questionnaire, parts 1 and 2), reported stress (measured with the perceive stress scale) and neuroticism (measured with the big five inventory short version). While state-anxiety, reported stress and neuroticism did not have a significant effect on efficiency, trait-anxiety had a significant main effect on efficiency (*F*(1,16) = 2.48, *p* = .048, *η*^2^ = 0.503) and interacted with difficulty (*F*(1,16) = 2.65, *p* = .037, *η*^2^ = 0.35). The main effect of trait-anxiety and its interaction with difficulty reached 22% and 25% power respectively. Post-hoc Pearson’s correlation tests revealed that participants with higher trait-anxiety were less efficient in the 2-back condition (*r* = -.24, *p* = .021) while this was not the case for the 0-back condition (*r* = .02, *p* = .85). Additionally, the triple interaction of trait-anxiety with difficulty and sound was significant (*F*(1,16) = 4, *p* = .006, *η*^2^ = 0.21). This effect reached 48% power. Trait-anxiety was negatively correlated with efficiency in the 2-back condition following startle sounds specifically (*r* = -.38, *p* = .034), but not control sounds (*r* = -.17, *p* = .37) (see Fig. 3).

### Brain activity

#### HbO

**Fig. 4:**
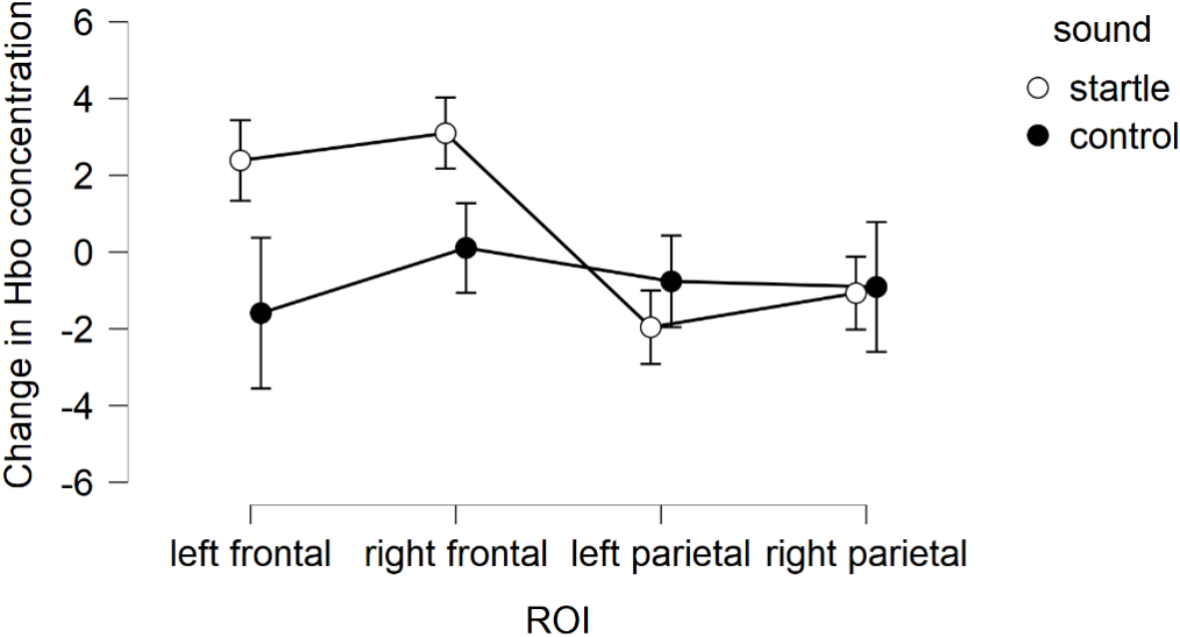
HbO changes during the TNT as a function of type of sound. Error bars represent the standard error of the mean.

 HbO concentration was not significantly different between the 0-back condition and the 2-back condition (*F*(1,26) = 1.33, *p* = .26, *η*^2^ = 0.008). Additionally, HbO was overall not significantly influenced by the type of sound (*F*(1,26) = 0.73, *p* = .40, *η*^2^ = 0.005) (Fig. 4). However, HbO concentration was significantly different between the ROI (*F*(3,78) = 4.07, *p* = .031, *η*^2^ = 0.013) and this further interacted with the sound condition (*F*(3,78) = 5.23, *p* = .01, *η*^2^ = 0.011). The sensitivity power analysis revealed an achieved power of 27% and 34% for the main effect of ROI and the interaction between ROI and sound type, respectively. As developed in what followed, activity following startle sounds was greater in the prefrontal cortex relative to the parietal cortex. More specifically, follow-up pairwise comparisons revealed that HbO concentration was significantly higher around the right frontal cortex than it was around the left parietal cortex (*t*(107) = 4.24, *p* < .001, *d* = 0.34), especially for startle sounds (*t*(53) = 5.81, *p* < .001, *d* = 0.67). Similarly, HbO concentration was also higher around the right frontal cortex than around the right parietal cortex (*t*(107) = 3.77, *p* < .001, *d* = 0.26), especially for startle sounds (*t*(53) = 4.45, *p* = .004, *d* = 0.54) as well as higher in the left frontal cortex than in the left parietal cortex following startle sounds (*t*(53) = 3.97, *p* = .01, *d* = 0.48). Additionally, HbO concentration was marginally higher following startle sounds relative to control sounds in the left frontal (*t*(53) = 1.81, *p* = .076, *d* = 0.33) and right frontal cortex (*t*(53) = 1.86, *p* = .068, *d* = 0.35), but not in the left parietal (*t*(53) = -0.78, *p* = .44, *d* = -0.13) or right parietal cortex (*t*(53) = -0.08, *p* = .94, *d* = -0.01). No other interactions reached significance.

#### HbR

HbR concentration was not significantly different between the 2-back and 0-back conditions (*F*(1,26) = 0.024, *p* = .88, *η*^2^ < 0.001) or between the startle and control sounds (*F*(1,26) = 0.11, *p* = .75, *η*^2^ < 0.001). The interaction between difficulty and type of sound was not significant (*F*(1,26) = 0.58, *p* = .45, *η*^2^ = 0.001). No other main effects or interactions reached significance.

### Face temperature

**Fig. 5:**
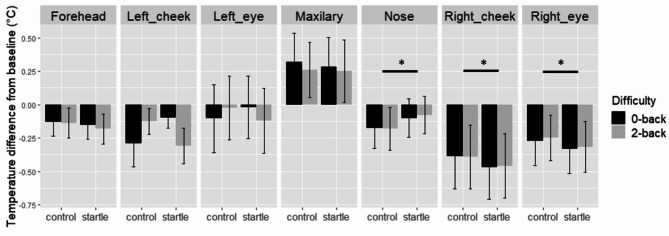
Mean temperature changes relative to baseline following sound onset in the different face regions.

We report below the analysis of facial temperature by region as a function of difficulty and sound condition (see Fig. 5).

#### Forehead

Task difficulty did not have a significant effect on the temperature of the forehead (*F*(1,24) = 0.08, *p* = .79, *η*^2^ < 0.001), nor did the sound condition (*F*(1,24) = 0.34, *p* = .56, *η*^2^ < 0.001). The interaction between difficulty and sound was not significant (*F*(1,24) = 1.75, *p* = .20, *η*^2^ = 0.001).

#### Nose

The temperature of the nosetip was not significantly different between the two difficulty conditions (*F*(1,26) = 2.1, *p* = .16, *η*^2^ < 0.001), but was significantly different between the startle and control sounds (*F*(1,26) = 5.5, *p* = .027, *η*^2^ = 0.006), with an increase in temperature after startle sounds relative to control sounds. The main effect of sound type achieved a power of 66%. The interaction between difficulty and sound was not significant (*F*(1,26) < 0.001, *p* = .99, *η*^2^ < 0.001).

#### Maxillary

The main effect of difficulty on temperature of the maxillary was not significant (*F*(1,20) = 0.05, *p* = .82, *η*^2^ < 0.001), nor was the main effect of sound (*F*(1,20) = 0.02, *p* = .90, *η*^2^ < 0.001). The interaction between difficulty and sound was not significant (*F*(1,20) = 0.08, *p* = .78, *η*^2^ < 0.001).

#### Left cheek

The main effect of difficulty on temperature of the left cheek was not significant (*F*(1,25) = 0.22, *p* = .65, *η*^2^ < 0.001), nor was the main effect of sound (*F*(1,25) = 0.001, *p* = .97, *η*^2^ < 0.001). The interaction between difficulty and sound was not significant (*F*(1,25) = 2.18, *p* = .15, *η*^2^ < 0.001).

#### Right cheek

The main effect of difficulty on temperature of the right cheek was not significant (*F*(1,26) = 0.13, *p* = .72, *η*^2^ < 0.001), but the main effect of sound was significant (*F*(1,26) = 7.54, *p* = .011, *η*^2^ = 0.006), with a decrease of temperature for startle sounds relative to control sounds. This effect achieved 78% power. The interaction between difficulty and sound was not significant (*F*(1,26) = 0.12, *p* = .73, *η*^2^ < 0.001).

Left eye: The temperature of the left eye was not significant influenced by difficulty (*F*(1,26) = 0.01, *p* = .91, *η*^2^ < 0.001), or sound (*F*(1,26) = 0.005, *p* = .94, *η*^2^ < 0.001). The interaction between difficulty and sound was not significant (*F*(1,26) = 1.25, *p* = .27, *η*^2^ < 0.001).

#### Right eye

Difficulty did not have a significant main effect on the right eye temperature (*F*(1,26) = 1.46, *p* = .24, *η*^2^ < 0.001), but the effect of sound was significant (*F*(1,26) = 4.71, *p* = .039, *η*^2^ = 0.001), with a decrease in temperature for startle sounds relative to control sounds. This effect achieved 58% power. The interaction between difficulty and sound was not significant (*F*(1,26) = 0.39, *p* = .54, *η*^2^ < 0.001).

## Discussion

In this study, we aimed to characterize the effects of startle on cognitive performance and physiological activity during a complex cognitive task. These laboratory conditions aimed at approaching what operators (e.g., aircraft pilots) may encounter when exposed to unexpected and stressful events during complex operations. Some of these events may trigger a startle response that can lead to a loss of cognitive capacity and even temporary cognitive incapacitation.

We found a rather paradoxical behavioral result: while we expected a degradation in performance following the startle stimuli, the opposite effect occurred, with a modest increase in performance in the 2-back condition (i.e., when the task was most difficult). This was associated with an increased brain response in the prefrontal cortex following startle (but not control sounds). Additionally, the temperature around some face landmarks showed differences between the startle and control stimuli.

While we designed the startle sounds to be threatening and expected them to induce acute stress and degrade performance, our results suggest that they may instead have acted as moderate stressors. We acknowledge that we did not measure participants’ subjective experience following startle sounds because of our experimental design (we only had them fill a questionnaire about the 2-back and 0-back conditions at the end of the experiment). But this idea is suggested by our behavioral observation of startle combined with the performance data. Contrary to acute stress that reduces working memory capacity^49,50^, the startle stimuli in the present study may have been insufficient to impair working memory and decrease performance at the TNT, and instead promoted increased effort and motivation. The literature indeed suggests that maintaining performance under stress is a trade-off between the engagement of more cognitive resources and less efficient information processing^51,52^. In line with the present findings, previous research found that individuals engaged in a cognitive task such as the TNT or other N-back tasks showed preserved, or even improved performance in presence of a stressor when they were under high (but not low) cognitive load^53,54^. As regards startle specifically, it should be noted that relatively few studies have investigated its effect on performance during high-load cognitive tasks as in the present study. While it is generally accepted that startle can interfere with all types of tasks^55,56^, the literature suggests a complex relationship between startle, cognitive performance and individual factors. On the one hand, cognitively demanding tasks have been found to reduce the effect of startle^57^ or to impact the processing of auditory stimuli (and potentially threatening sounds), a phenomenon labeled as inattentional deafness^58^. On the other hand, the effect of startle on performance can be amplified by the type of attention orientation during a task (e.g., self-oriented attention)^59^. In light of the literature above and the Processing Efficiency Theory^51^, we suggest that the unpleasant startling sounds have triggered moderate stress in participants, leading to increased motivation and effort. This may have increased the resources engaged during the most difficult condition of the task to cope with task demand.

We should however mention some limitations of our study: since participants were in a lab setting and the startle sounds were repeated 6 times, we may assume that only the first startle sound may have been really threatening, and the subsequent ones may have been more expected. Despite this limitation and the presumably low arousal temporary triggered by the startle sound, our investigations of individual differences revealed an interesting association between trait-anxiety and performance. Higher trait-anxiety was associated with decreased performance, especially under high cognitive load and following startle. Although we lack statistical power for this result, this suggests that higher proneness to anxiety may have enhanced the startle response and limited the recruitment of cognitive resources following the startle sound to meet task demand. This hypothesis is in keeping with previous evidence of increased startle response in individuals with higher trait-anxiety^60,61^, though other studies found no relationship between the intensity of startle and trait-anxiety^62,63^. We note, however, that we did not observe an effect of neuroticism on performance at this cognitively demanding task following startle, similarly to what has been reported at a less cognitively demanding task^64^. We should however keep in mind that other individual factors beyond anxiety or personality traits, such as the tolerance of uncertainty^65^, may also influence people’s reaction following startle.

The idea that participants engaged more cognitive resources after startle to maintain a high level of performance is supported by our brain activity results. Indeed, we observed an increase in prefrontal cortex activity after the startle stimuli, particularly when the task was difficult. Activity in the (right) DLPFC, which is part of the ECN and one of the brain regions most sensitive to stressors, may reflect cognitive control processes to manage a stressor/distractor^66,67^. Yet, the relationship between stress and DLPFC activity may depend on the type of stressor. On the one hand, acute stress is associated with a shift of brain activity from ECN to the salience network^68,69^. More specifically, it is often assumed that acute stress reduces DLFPC activity, leading to impaired WM capacity^69,70^, whereas artificial stimulation of the DLPFC counters the effect of stress on WM^71^. On the other hand, increased recruitment of the prefrontal cortex has also been found in response to (more moderate) stressors, and this was associated with adequate task performance^22,23,53^. The present study echoes with this line of research, suggesting that the startle stimuli led to increased prefrontal activity to support cognitive control and maintain task performance. We view this difference in prefrontal activity as compensatory, instead of reflecting enhanced attention following startle. If startle were actually to enhance overall attention, this effect would primarily concern the source of stress (attention orientation toward the startle sound), which would subsequently degrade performance following startle stimuli. This was however not the case. We should however remain cautious in the interpretation of the brain activity results since we had low statistical power associated with the observed effects.

In an effort to better capture the physiological response to startle, we included measures of face temperature with the assumption that they could complement measures of brain activity. The use of fITI to track changes in cognitive and/or affective states is relatively recent and few studies have shown how startle affects face temperature, with a large variability between studies in the face areas targeted. Although we found an effect of startle on a few face areas (3 out of 7 targets), our pattern of temperature variation is rather hard to interpret in light of the mixed evidence in the literature. For instance, there is evidence for a decrease in the nose temperature following startle or surprise^44^, in contrast to the effect we observed in this area. In parallel, previous work shows a decrease in the temperature of the cheeks^40,44^, in line with the present results, or an increase in the temperature of the maxillary and forehead^42,45^, an effect not observed in the present study. A limitation we may object to our fITI data acquisition is that this technique requires very controlled conditions to account for temperature variation depending on circadian rhythms or participants’ previous activities (intense exercise, consumption of psychoactive substance) because of their potential impact on body (and face) temperature. In the present study, we refrained from giving participants too strict instructions to facilitate our recruitment effort and collected data at different times of the day. Even though we used a within-subjects design and baseline-corrected face temperature in the targeted areas, we cannot exclude that natural temperature variation between participants may have added noise to the data and thus masked the effect of our experimental manipulation. However challenging the implementation of fITI currently is, attempts to use this tool in more natural settings are on the rise. Despite its limitations, we believe that the implementation of fITI in future research in association with non-invasive brain imaging tools may offer interesting perspectives in Neuroergonomics.

## Methods

### Participants

Thirty-four participants completed the study (age = 24.8 years ± 4.3, 7 females). Most of them were local students with at least two years of Higher Education. Participants reported normal audition and normal or corrected-to-normal vision. None wore glasses during the session. All participants gave their written informed consent before the start of the experiment. All experimental procedures were performed in accordance with relevant guidelines and regulations and were approved by the local ethics committee (Comité d’éthique sur la recherche de l’université fédérale Toulouse – Midi Pyrénées, IRB # 00011835 − 2023 − 0918 − 681).

## Material

### Mental arithmetic N-back task

**Fig. 6:**
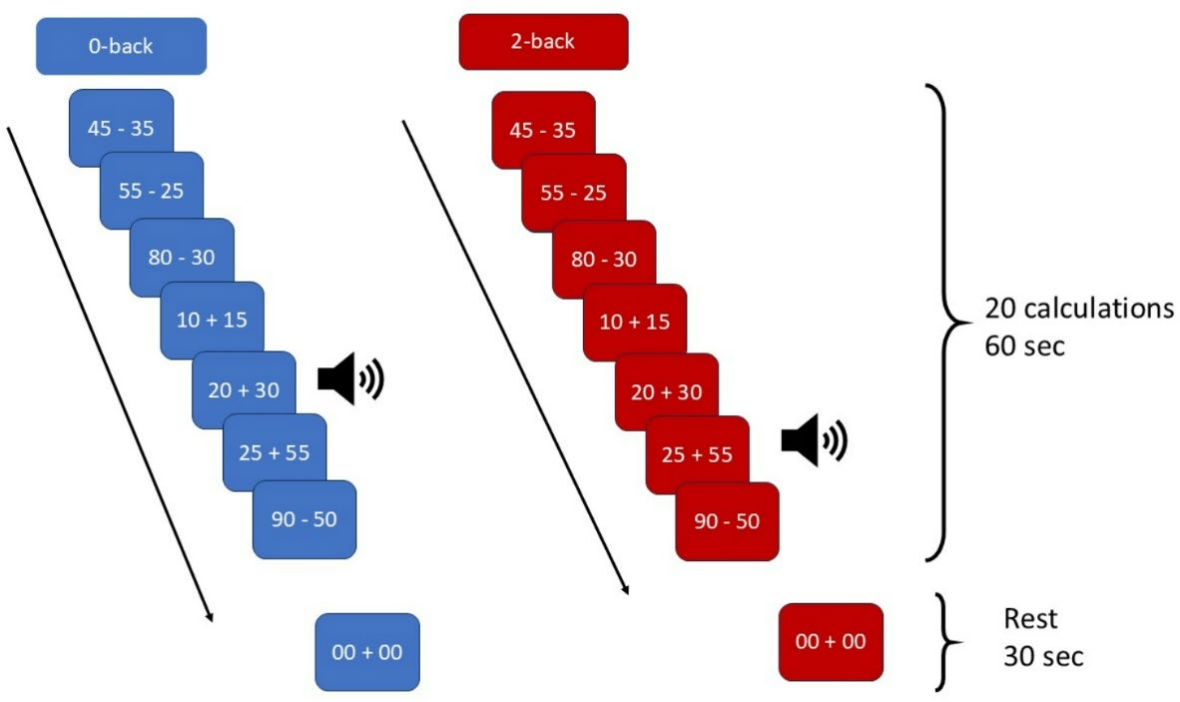
Toulouse N-back task (TNT) comprising 60-second blocks of mental calculation alternating between the 0-back and the 2-back condition (14 blocks total) ending with a 30-second rest period.

Participants completed an adaptation of the N-back task known as the Toulouse N-back task (TNT)^23^. In addition to comparing items between trials as in the traditional N-back task, participants also performed mental arithmetic (Fig. 6). More specifically, in the 0-back condition, participants computed the result of an arithmetic operation and compared it to a reference number (i.e., 50). In the 2-back condition, participants computed the result of a calculation and had to respond whether it matched the result of the calculation presented two trials earlier. Therefore, memory load was higher for the 2-back condition. Calculations consisted of additions and subtractions of double-digit numbers which were multiples of 5 (e.g., 15 + 30; 80 − 35). There were 7 blocks of the 0-back condition alternating with 7 blocks of the 2-back condition. Each block lasted 60 s and included 20 calculations presented for 2 s with a 1-second inter-stimulus interval (note that no response was expected for the first two trials of the 2-back blocks). Responses were provided with a 2-button Cedrus response pad (RB-740, Cedrus Corporation, San Pedro, CA). Each block ended with a short rest period featuring the same string (“00 + 00”) that did not require participants to provide any response. Importantly, brief sounds were randomly presented during the TNT to induce startle (see Procedure). The task was implemented in Matlab (MathWorks) using Psychtoolbox 3^72–74^.

### Sound stimuli

We aimed at inducing a startle reflex using unpredictable, loud, and unusual sounds. Two stimuli were selected from an initial pool of six pretested sounds based on their effectiveness at eliciting startle. These stimuli consisted of a white noise and a recording of a metallic bar falling on the ground with an intensity of 95dB. We additionally used a soft and continuous beep as a control sound with an intensity of 65dB. All three acoustic stimuli lasted 1000 ms.

### Self-report questionnaires

Immediately before or after the TNT, participants completed several self-report questionnaires assessing their impression of the TNT, personality traits, emotion regulation, proneness to anxiety, and stimulating substance intake (coffee, alcohol, …) in the last 24 h.

*Nasa Task load index*^75^. Participants rated how demanding they found each condition of the TNT in terms of mental effort, temporal demand, physical effort, performance and frustration.

*Big Five Personality Inventory* short version^76^ (BFI 10). This questionnaire evaluates five dimensions of personality (openness to experience, conscientiousness, extraversion, agreeableness, and neuroticism) in 10 items (2 items per personality dimension).

*Multidimensional Emotion Questionnaire*^77^ (MEQ). Participants evaluate how often they feel each of 10 different emotions, how intense and for how long they generally feel it, and how easy it is to regulate.

*State Anxiety Inventory* - Trait and State^78,79^ (STAI). This questionnaire assesses participants’ anxiety level in 40 items. The first 20 items measuring state anxiety were administered before the TNT while the remaining 20 items measuring trait anxiety were administered after the TNT.

*Perceived stress scale*^80^ (PSS 14). In 14 items, this questionnaire measures how people deal with daily-life stress.

## Procedure

### Experimental set-up

**Fig. 7:**
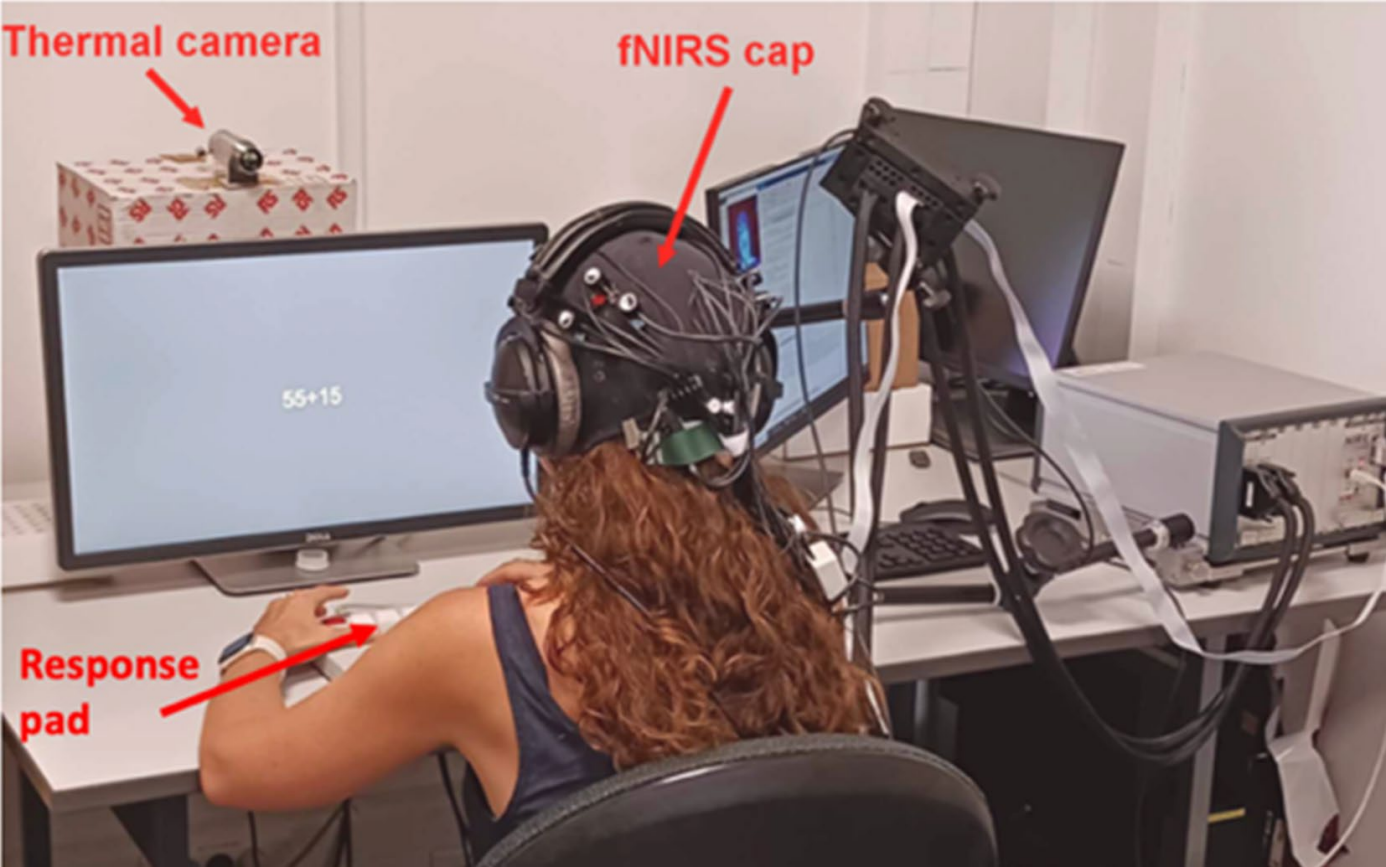
Illustration of the experimental setup. The TNT task is displayed on the screen.

After filling in a few questionnaires, participants were familiarized with the TNT. They completed a short training featuring one block of each difficulty level (0-back and 2-back). They were then instructed to stay as focused as possible during the task and to respond as quickly and accurately as possible. They were also told to avoid moving their head. They were equipped with a headset and told that it was meant to maintain the NIRS optic fibers/cables. The thermal camera was placed in front of participants at a distance of 1 m. The occurrence of sounds was not mentioned to participants. The TNT started with a 2-minute baseline (blank screen) and lasted 25 min. See Fig. 7 for an illustration of the experimental set-up.

### Randomization

Participants started with either the 2-back or the 0-back condition (pseudo-randomized). During the TNT, the sound stimuli were presented pseudo-randomly during the calculation blocks according to the following conditions: First, there was a total of 10 sound occurrences. The startling sounds (metallic bar and white noise) were played 3 times each and the control sound (continuous beep) was played 4 times. The imbalanced number of startle sounds versus control sounds was intentional, as we limited the number of repetitions of the control sound to limit the experiment duration. Although this may have contributed to differences in the signal-to-noise ratio between conditions, such imbalances are common, for example in oddball paradigms^81,82^.

Second, no more than one sound was presented per calculation block and some calculation blocks did not include any sound. Third, the sequence of sounds was randomized (i.e., some participants were first presented with a startling sound while others were first presented with a control sound) but each type of sound (control or startle) was played once or twice in the 0-back and 2-back conditions. Fourth, sound onset was pseudo-randomized so that the sound was played between 7 s and 30 s after the beginning of the block. Once participants completed the TNT, they filled out the remaining online questionnaires.

### fNIRS data acquisition

**Fig. 8:**
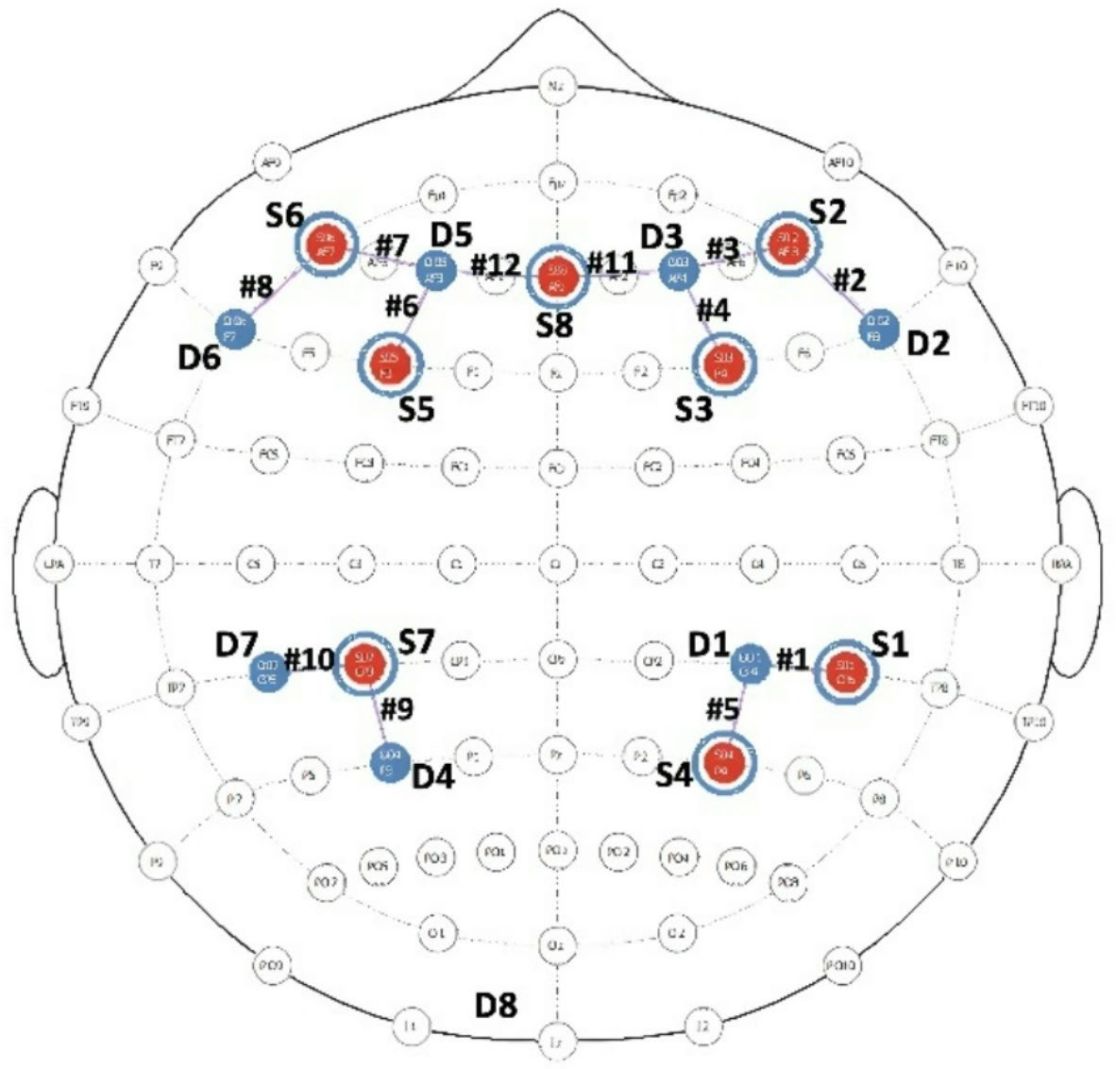
fNIRS montage.

Brain activity was recorded via the NIRScout system and the NIRStar 14.3 software (NIRx Medical Technologies, LLC. Los Angeles, California). The system emitted 2-wavelength of infrared light (850 nm and 760 nm) at a sampling rate of 7.8 Hz to assess oxy- and deoxygenated hemoglobin blood concentration, respectively. The headcap was equipped with 15 optodes, including 8 sources and 7 detectors. The source-detector distance was set to 3 cm for adjacent optodes. Optodes were positioned according to the 10–10 international system in such a way that the montage included 12 channels (see Fig. 8), 8 of them covering the bilateral frontal cortex and 4 others covering the bilateral parietal cortex. Additionally, 8 short-distance channels were used to control for confounding signals (e.g., cardiovascular artifacts and scalp perfusion).

### Thermal imaging data acquisition

Face temperature was acquired via an Optris Xi400 infrared camera (Optris Infrared Sensing LLC, Portsmouth, USA), with an optical resolution of 382 × 288 pixels, a temperature range in the − 20 °C to 900 °C, a thermal sensitivity of 80mK, and a maximum sampling rate of 35 Hz. The experimentation room was maintained at a constant temperature of 22 °C, did not have natural light and a fixed light source was positioned far from the camera.

## Analysis

### Behavioral analysis

First, perceived task demand as assessed by the NASA TLX was compared between the two difficulty conditions. Because the data did not follow the normal distribution, we used a bilateral Wilcoxon signed-rank test on each of the 6 dimensions included in this self-report questionnaire. Note that these analyses were run on 31 participants because of missing data for four participants. An a-posteriori sensitivity power analysis was conducted in G*power to determine the achieved power associated with the observed effect under these assumptions (Wilcoxon signed-rank test matched pairs, two-tailed test, parent distribution = normal, α = 0.05, *N* = 31).

Second, performance at the TNT was analyzed as a function of task difficulty (0-back vs. 2-back) and sound type. Given that startle sounds were expected to impact performance only briefly, we selected only the first three calculations following each sound onset. We computed the efficiency score (accuracy / rt) and submitted the mean efficiency score to statistical analysis. As the conditions to conduct parametric testing were met, we analyzed efficiency using a 2-way repeated-measures ANOVA with sound type and difficulty as within-subject factors. This analysis was run on 34 participants. We report the general *η*^2^ in the result section as the estimate of effect size. A post-hoc sensitivity power analysis was conducted under the following assumptions to determine the statistical power associated with the effect size observed (ANOVA: Fixed effects, special, main effects and interaction; α = 0.05; *N* = 34; numerator degree of freedom = 1, 4 groups).

In exploratory analysis, we tested whether participants’ efficiency at the TNT was related to their anxiety level, perceived stress or neuroticism. To this aim, we conducted a repeated-measures ANOVA on efficiency as a function of difficulty and sound and with score at the corresponding test as a covariate and using the same parameters as above, except that (i) the α threshold was set to 0.007 to adjust for multiple testing (7 separate tests) and (ii) the number of participants was adjusted based on data availability for each personality questionnaire.

### fNIRS data analysis

Preprocessing. Data was analyzed using the MNE software (version 0.6) implemented in python version 3.11. First, light intensity was converted to optical density for both chromophores. The signal from the short channels was regressed out. Optical density was then converted to concentration using the modified Beer-Lambert law (ppf = 0.1). Channels with a scalp coupling index (as implemented in MNE) lower than 0.5 were flagged as bad and interpolated. The average number of interpolated channels in the final sample was 1 (min = 0, max = 3). Five participants were excluded because of low data quality and two additional participants were excluded due to technical issues. We used a temporal derivative distribution repair procedure implemented in MNE to correct for motion artifacts. Data were then low-pass filtered at 0.7 Hz and high-pass filtered at 0.02 Hz.

Individual-level analysis. Next, the preprocessed signal was segmented into epochs for each sound category by difficulty condition (4 combinations: startle 0-back, startle 2-back, control 0-back, control 2-back). All epochs were defined as a 15-second time window with onset time-locked to the sound onset and a baseline defined as the 5-second period preceding sound onset (Note that this time window aligns with a previous study on startle^83^, showing a short-term change in physiological markers following a startle stimulus which resolved approximately 10–15 s later, reflecting a short and acute stress reaction.) In individual-level analyses, the mean HbO and HbR concentration was averaged across trials of the same epoch type (startle 0-back, startle 2-back, control 0-back, control 2-back). Next, regions of interest (ROI) were defined based on the 12 channels of the fNIRS montage to analyze the signal as a function of laterality (left vs. right hemisphere) and anteriority (frontal vs. parietal), leading to 4 ROIs: left frontal, left parietal, right frontal, right parietal. The mean signal resulting from epoching was thus averaged within each ROI for each chromophore and subject and then submitted to group-level inferential statistics. The final sample included 27 participants.

Group-level analysis. Mean HbO and HbR concentrations were analyzed using a repeated-measures ANOVA with task difficulty, sound category, and ROI as within-subject factors. The Greenhouse-Geiser correction was used when the assumption of sphericity was violated. We then conducted a sensitivity power analysis to determine the power reached for the main effect of ROI on HbO and the interaction between ROI and sound (ANOVA: Fixed effects, special, main effects and interaction; α = 0.05; *N* = 27; numerator df = 3; number of groups = 16).

### Thermal imaging data analysis

**Fig. 9:**
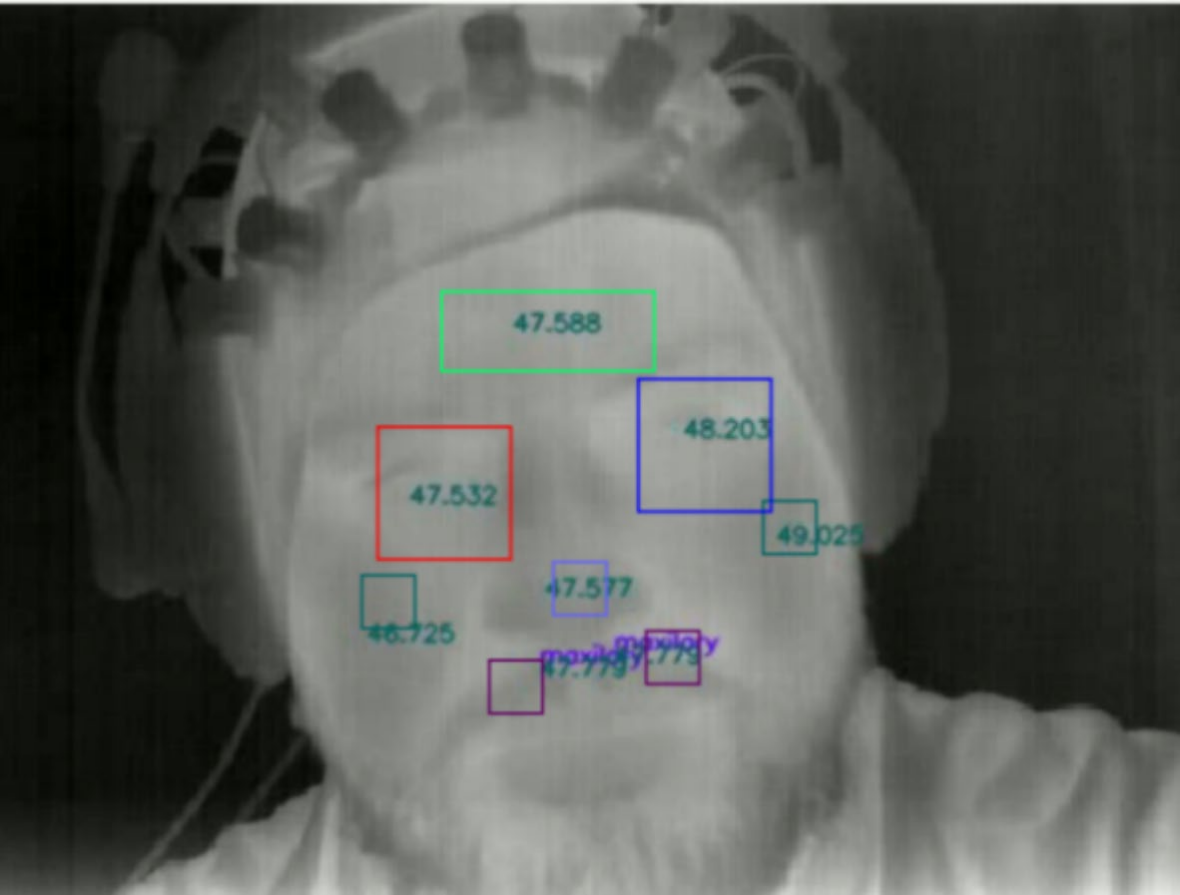
Face landmarks and their surrounding areas in which temperature changes from baseline were averaged.

In order to run facial recognition on relevant time windows, we segmented the session into short videos, corresponding to the period following each of the 10 sounds presented to subjects in addition to the baseline. We generated 30-second videos with the onset time-locked to the sound presentation as well as a 120-second video with onset time-locked to the baseline onset and offset time-locked to the baseline offset. We generated black-and-white videos instead of color videos because doing so improved the accuracy of facial recognition. These videos were then submitted to a facial landmark detection algorithm from MediaPipe (https://ai.google.dev/edge/mediapipe/solutions/vision/face_landmarker) implemented in python. To sum up, this technique allowed to retrieve the temperature value at the specified face landmarks for every frame. We targeted several landmarks (see Fig. 9) whose temperature variation has been associated with variation in cognitive and/or affective states (Ioannou et al., 2014), namely the forehead (mediapipe face mesh index 151), left and right cheeks (mediapipe face mesh indices 50 and 280), nosetip (mediapipe face mesh index 1), left and right eyes (mediapipe face mesh indices 133,33 and 362,263) and maxillary (mediapipe face mesh indices 167, 393). The minimum detection confidence and minimum tracking confidence parameters were initially set to 0 and then adjusted for a few participants when this improved detection and tracking of facial landmarks.

Regions of interest were then drawn to analyze temperature variation on a set of pixels surrounding a landmark. To this aim, we used a large square box for the periorbital area, a large rectangular box for the forehead, and a small square box for the maxilary, cheeks and nosetip. In cases of landmarks not positioned correctly but whose incorrect position was consistent across frames, the ROI was manually repositioned on a video-by-video basis. Subjects whose most landmarks were not recognized correctly on most videos or positioned inconsistently were excluded from the analysis (*N* = 4). An additional participant was excluded from the analysis due to technical issue (*N* = 1). Therefore, the statistical analyses were run on 29 participants, 23 of whom were included in the fNIRS data analysis.

Next, temperature values associated with the pixels were averaged within a given ROI and across frames for each video. Finally, the mean temperature at each ROI was averaged across subjects and submitted to statistical analysis. Because the data were normally distributed in all face landmarks, we analyzed the mean temperature change with a repeated-measures ANOVA as a function of the difficulty condition (2 back versus 0 back) and type of sound (startle versus control). A sensitivity power analysis was conducted in G*power with the following assumptions and parameters: ANOVA: Fixed effects, special, main effects and interaction; α = 0.05, *N* = 29 (note that the sample size was adjusted for each ROI); numerator df = 1; 4 groups).

## Electronic supplementary material

Below is the link to the electronic supplementary material.

Supplementary Material 1

## Data Availability

The data can be found at the following link: https://osf.io/kav3p/?view_only=d0bad04fab9d423d9bfb078d3bb56a9e.
