# Supplementary material for: Effects of startle on cognitive performance and physiological activity revealed by fNIRS and thermal imaging
Source: Sci Rep. 2025 Feb 26;15:6878. doi: 10.1038/s41598-025-90540-z (PMC11865621; doi:10.1038/s41598-025-90540-z)
Supplement: Supplementary file 1 — Supplementary Material 1 [file 41598_2025_90540_MOESM1_ESM.docx]

**S1. Accuracy and response times at the TNT**

As with the analysis of efficiency, we analyzed accuracy and responses times at the TNT as a function of the within-subject factors difficulty and sound type.

As for accuracy, difficulty had a significant effect on accuracy (*F*(1,33) = 91, *p* < .001, *ր²*  = 0.39) and interacted with sound type (*F*(1,33) = 6.7, *p* = .014, *ր²*  = 0.025). The effect of sound was not significant (*F*(1,33) = 0.16, *p* = .69, *ր²* < 0.001). Post-hoc pairwise contrasts revealed that participants tended to be more accurate following startle relative to control sounds for the 2-back condition (*t*(33) = -1.7, *p* = .097) but less accurate for startle relative to control sounds in the 0-back condition (*t*(33) = 1.97, *p* = .057). See Supplementary Figure 1 below.

As for response times, both difficulty (*F*(1,33) = 14.5, *p* < .001, *ր²*  = 0.11) and sound type (*F*(1,33) = 4.33, *p* = .045, *ր²*  = 0.023) had a significant effect. The interaction between difficulty and sound type was significant (*F*(1,33) = 4.95, *p* = .033, *ր²*  = 0.021). Post-hoc pairwise contrasts revealed that participants were faster to respond following startle sound relative to control sound in the 2-back condition (*t*(33) = 2.31, *p* = .027), but this was not the case in the 0-back condition (*t*(33) = 0.14, *p* = .89). See Supplementary Figure 2 below.

In brief, our behavioral data show that a combination of response time and accuracy contributes to differences in performance following startle sounds relative to control sounds depending on the difficulty condition.


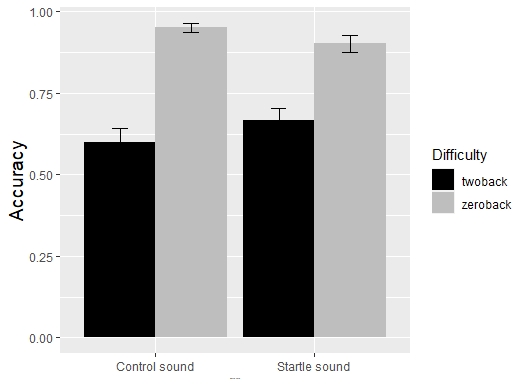


Supplementary Figure 1. Accuracy at the TNT as a function of difficulty and sound type.


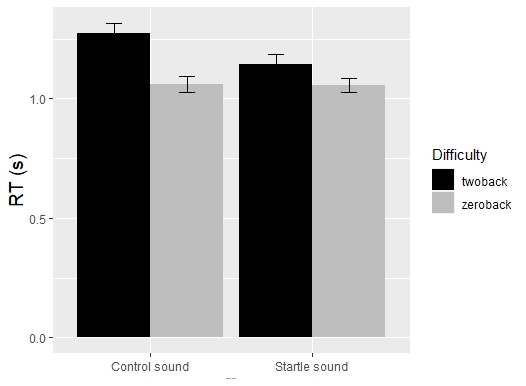


Supplementary Figure 2. Mean response time at the TNT as a function of difficulty and sound type.
